# A factor analysis of posttraumatic stress disorder symptoms using data pooled from two venlafaxine extended-release clinical trials

**DOI:** 10.1002/brb3.183

**Published:** 2013-10-16

**Authors:** Dan J Stein, Barbara O Rothbaum, David S Baldwin, Annette Szumski, Ronald Pedersen, Jonathan R T Davidson

**Affiliations:** 1 University of Cape TownCape Town, South Africa; 2 Emory University School of MedicineAtlanta, Georgia; 3 Faculty of Medicine, University of SouthamptonHampshire, U.K; 4 Pfizer Inc formerly Wyeth ResearchCollegeville, Pennsylvania; 5 Duke University Medical CenterDurham, North Carolina

**Keywords:** CAPS-SX_17_, DSM, factor analysis, posttraumatic stress disorder, venlafaxine

## Abstract

**Background:**

Confirmatory factor analysis (CFA) of *Diagnostic and Statistical Manual of Mental Disorders* (Fourth Edition) (DSM-IV) three-factor posttraumatic stress disorder (PTSD) diagnostic criteria was conducted to determine fit for this patient population. An exploratory factor analysis (EFA) of alternate symptom structures was planned to identify symptoms that cluster in this population. The response of symptom factors to treatment with venlafaxine extended release (ER) was explored.

**Methods:**

Baseline 17-item Clinician-Administered PTSD Scale (CAPS-SX_17_) data were pooled from patients enrolled in two double-blind, randomized, placebo-controlled trials. The CFA was conducted using maximum likelihood and weighted, least-squares factor extraction methods. The EFA was performed using a polychoric correlation covariance matrix and Pearson correlation matrix.

**Results:**

Data from a pooled population of 685 patients (venlafaxine ER: *n* = 339; placebo: *n* = 346) were analyzed. CFA rejected the DSM-IV three-factor structure. The EFA identified a different three-factor structure as the best fit: factor 1 included reexperiencing symptoms, factor 2 included symptoms of altered mood and cognition, whereas factor 3 comprised avoidance and arousal symptoms. All DSM-IV symptom factors and all factors in the identified three-factor model responded positively to venlafaxine ER treatment.

**Conclusions:**

Data are consistent with literature failing to confirm the three-factor structure of DSM-IV PTSD, and they support the DSM-5 inclusion of a symptom cluster addressing altered mood and cognition in PTSD. The efficacy of venlafaxine ER in reducing a range of symptom clusters in PTSD is consistent with its multiple mechanisms of action.

## Introduction

Posttraumatic stress disorder (PTSD) is characterized by a broad range of symptoms and behaviors stemming from exposure to a traumatic event that is a perceived threat to oneself or others. The PTSD symptoms described in the *Diagnostic and Statistical Manual of Mental Disorders* (Fourth Edition) (DSM-IV) (American Psychiatric Association 1994) are divided into three clusters: reexperiencing, avoidance/numbing, and hyperarousal. The validity of the current conceptualization of PTSD described in DSM-IV has been questioned because of the often heterogeneous presentation of PTSD; the overlap in symptom criteria between PTSD, other anxiety disorders, and major depressive disorder; and the high comorbidity rate among these disorders (North et al. 2009). A number of factor analyses have been conducted, most suggesting alternative two-, three-, or four-factor models of PTSD that provide different conceptualizations of PTSD: including additional symptom clusters such as dysphoria, or distinguishing between an active avoidance and passive numbing factor (Foa et al. 1995; Buckley et al. 1998; King et al. 1998; Asmundson et al. 2000; Amdur and Liberzon 2001; Gaffney 2003; Baschnagel et al. 2005; Elhai et al. 2009).

Posttraumatic stress disorder factor analyses traditionally have focused only on identifying symptoms that cluster in a given population, while significantly less attention has been paid to exploring how these factors respond to treatment. Antidepressant pharmacotherapy has been shown to be clinically efficacious for treating PTSD (Davidson 2006). However, inconsistencies in patterns of treatment response, including variations in response rates (Stein et al. 2009), have been observed in PTSD patients treated with these agents. By assessing the relationship between PTSD symptom clusters and response to pharmacotherapy, we may further our ability to predict response to treatment and possibly contribute to our understanding of the way in which these treatments ameliorate PTSD symptomatology. Analogous studies in other anxiety disorders have been of value (Mataix-Cols et al. 1999; Stein et al. 2006a, 2007).

This factor analysis was designed to investigate PTSD symptom clusters pooled from patients who participated in two randomized, placebo-controlled clinical trials that demonstrated the efficacy of flexible doses of venlafaxine extended release (ER) (37.5–300 mg/d) for the treatment of PTSD (Davidson et al. 2006a,b). The venlafaxine ER PTSD data set provides the opportunity to conduct a factor analysis using a large cross-national sample and to assess how the identified symptom clusters respond to treatment with venlafaxine ER. Our hope was that these analyses would shed additional light not only on the general question of the symptom structure of PTSD but also on the more specific question of whether PTSD symptom clusters are responsive to venlafaxine treatment.

## Methods

### Study design

Baseline and week 12 CAPS-SX_17_ data from two double-blind, randomized, placebo-controlled trials that assessed the efficacy of venlafaxine ER for the treatment of PTSD were pooled for these factor analyses. The full methodology and results of these studies have been published elsewhere (Davidson et al. 2006a,b). The first was a 12-week study, conducted in the US, that assessed the efficacy of venlafaxine ER (37.5–300 mg/d) and sertraline (25–200 mg/d), versus placebo for treating PTSD (Davidson et al. 2006b). The second study was 24 weeks in duration and conducted in 12 countries: Argentina, Chile, Colombia, Denmark, Finland, Mexico, Norway, Portugal, South Africa, Spain, Sweden, and the United Kingdom. It was designed to compare the efficacy of venlafaxine ER (37.5–300 mg/d) with placebo (Davidson et al. 2006a). For both studies, the dosing schedule for venlafaxine ER was flexible and could be increased to a maximum of 75 mg/d at day 5, 150 mg/d at day 14, 225 mg/d at day 28, and 300 mg/d at day 42. These studies were conducted in accordance with the US Food and Drug Administration Code of Federal Regulations (21CFR, Part 50), with the ethical principles in the Declaration of Helsinki, and were consistent with Good Clinical Practice and applicable regulatory requirements. They received independent ethics or institutional review board approval in each country before the study began, and written informed consent was obtained from all patients prior to enrollment. For the current factor analyses, only data from the venlafaxine ER and placebo groups from this study were included.

### Patients

Study participants were medically stable adult outpatients with a primary diagnosis of PTSD according to DSM-IV criteria, who had been experiencing symptoms for ≥6 months and had a baseline score of ≥60 on the 17-item Clinician-Administered PTSD Scale (CAPS-SX_17_) (Blake et al. 1995). Exclusion criteria included a current primary diagnosis of major depression or an anxiety disorder other than PTSD; a current mental disorder due to a general medical condition or history of bipolar disorder, schizophrenia, or other psychotic disorder; alcohol or drug abuse or dependence within 6 months of randomization or a positive urine drug screen; and a high risk of suicide or violence. The baseline demographic characteristics for the individual studies and the pooled population are described in Table 1.

**Table 1:** Baseline demographic characteristics

	Study 1		Study 2		Pooled	
			
	Venlafaxine ER (*n* = 179)	Placebo (*n* = 179)	Venlafaxine ER (*n* = 161)	Placebo (*n* = 168)	Venlafaxine ER (*n* = 340)	Placebo (*n* = 347)
Race, *n* (%)						
White	121 (67.6)	135 (75.4)	92 (57.1)	100 (59.5)	213 (62.7)	235 (67.7)
Black	36 (20.1)	21 (11.7)	4 (2.5)	3 (1.8)	40 (11.8)	24 (6.9)
Hispanic	20 (11.2)	17 (9.5)	54 (33.5)	57 (33.9)	74 (21.8)	74 (21.3)
Asian	0 (0.0)	0 (0.0)	1 (0.6)	2 (1.2)	1 (0.3)	2 (0.6)
Other	2 (1.1)	6 (3.4)	10 (6.2)	6 (3.6)	12 (3.5)	12 (3.5)
Gender, *n* (%)						
Female	124 (69.3)	114 (63.7)	89 (55.3)	89 (53.0)	213 (62.7)	203 (58.5)
Male	55 (30.7)	65 (36.3)	72 (44.7)	79 (47.0)	127 (37.4)	144 (41.5)
Type, *n* (%)						
Accidental injury	18 (10.1)	21 (11.7)	30 (18.6)	31 (18.5)	48 (14.1)	52 (15.0)
Combat	19 (10.6)	18 (10.1)	20 (12.4)	20 (11.9)	39 (11.5)	38 (11.0)
Natural disaster	2 (1.1)	0 (0.0)	5 (3.1)	2 (1.2)	7 (2.1)	2 (0.6)
Nonsexual abuse	51 (28.5)	48 (26.8)	42 (26.1)	52 (30.1)	93 (27.4)	100 (28.8)
Sexual abuse (adult)	26 (14.5)	26 (14.5)	19 (11.8)	21 (12.5)	45 (13.2)	47 (13.5)
Sexual abuse (childhood)	28 (15.6)	28 (15.6)	2 (1.2)	1 (0.6)	30 (8.8)	29 (8.4)
Unexpected death	22 (12.3)	21 (11.7)	26 (16.2)	18 (10.7)	48 (14.1)	39 (11.2)
Unknown	3 (1.7)	2 (1.1)	0 (0.0)	1 (0.6)	3 (0.9)	3 (0.9)
Witnessing	7 (3.9)	11 (6.2)	11 (6.8)	13 (7.7)	18 (5.3)	24 (6.9)
Other	3 (1.7)	4 (2.2)	6 (3.7)	9 (5.4)	9 (2.7)	13 (3.8)
CAPsS-SX_17_, mean (SD)						
Total	84.0 (15.0)	81.6 (14.7)	81.0 (14.6)	82.9 (15.5)	82.6 (14.8)	82.2 (15.1)

CAPS-SX_17_, 17-item Clinician-Administered PTSD Scale; ER, extended release; PTSD, posttraumatic stress disorder.

### Outcomes

The CAPS-SX_17_ was the primary outcome measure for both studies. The CAPS-SX_17_ is a rating scale based on the 17 PTSD symptoms described in DSM-IV (Table 2), which includes three clusters or subscales (i.e., reexperiencing, avoidance/numbing, and hyperarousal).

**Table 2:** DSM-IV/CAPS-SX_17_ PTSD symptom clusters (the prespecified three-factor structure)

Reexperiencing	Item 1: Intrusive recollections
	Item 2: Distressing dreams
	Item 3: Feeling events were recurring
	Item 4: Distress at exposure to cues
	Item 5: Reactivity on exposure to cues
Avoidance/Numbing	Item 6: Avoidance of thoughts, feelings, or conversations
	Item 7: Avoidance of activities, places, or people
	Item 8: Inability to recall important aspects of trauma
	Item 9: Diminished interest or participation in activities
	Item 10: Detachment or estrangement
	Item 11: Restricted range of affect
	Item 12: Sense of a foreshortened future
Hyperarousal	Item 13: Difficulty falling or staying asleep
	Item 14: Irritability or outbursts of anger
	Item 15: Difficulty concentrating
	Item 16: Hypervigilance
	Item 17: Exaggerated startle response

CAPS-SX_17_, 17-item Clinician-Administered PTSD Scale; DSM-IV, *Diagnostic and Statistical Manual of Mental Disorders* (Fourth Edition); PTSD, posttraumatic stress disorder.

### Statistical analysis

#### Factor analyses

These factor analyses were performed using baseline data collected prior to treatment administration, which allowed for the pooling of the venlafaxine ER and placebo treatment arms of both studies. Additionally, separate analyses of each individual study were conducted as a means of cross-validation. An initial confirmatory factor analysis (CFA) was performed using the prespecified three-factor structure described in the DSM-IV to determine whether the current data fit this structure. If the data did not fit, an exploratory factor analysis (EFA) was planned to identify symptoms that cluster in this population and to assess how these factors respond to treatment.

The CFA was performed using a maximum likelihood factor extraction method for normally distributed data and a weighted least-squares factor extraction method for categorical data; two methods were used to see if similar factors were extracted with both methods. These CFA models used Hu and Bentler's (1999) recommendation of a combination of two goodness-of-fit indexes (Hu and Bentler 1999). This combination included a noncentrality-based index such as a root mean square error of approximation (RMSEA) to indicate the amount of unexplained variance with a criteria of <0.60, and a relative fit index, such as Bentler–Bonett Non-normed Index that has a penalty for adding parameters with a criteria of >0.90 for acceptable fit.

The EFA was performed using a polychoric correlation covariance matrix; a technique for estimating correlations among theorized normally distributed continuous latent variables from observed ordinal variables. A sensitivity analysis was conducted that used the Pearson correlation matrix. The maximum likelihood extraction method was used to extract the factors, and an oblique, promax factor rotation method was used to allow for correlated factors. The maximum likelihood factor extraction method, which provides statistical testing (i.e., goodness of fit for the model, significance testing of factor loadings), is best for relatively normally distributed data (Fabrigar et al. 1997). The number of extracted factors to retain was determined by examining scree plots of factors versus eigenvalues, Horn's parallel analysis, and the Schwarz's Bayesian Criteria (SBC) goodness-of-fit test (Fabrigar et al. 1999). To determine whether an item belonged in a factor, the lower limit of the 95% confidence interval (CI) for that item was required to be greater than 0.30 in either study individually or in the pooled study analysis.

### Treatment effect analysis

The treatment effect analysis was conducted using adjusted effect sizes from an analysis of covariance (ANCOVA) model of change from baseline to week 12 using unit-standardized CAPS-SX_17_ scores and unit-standardized, factor-transformed CAPS-SX_17_ scores. CAPS-SX_17_ scores were standardized by dividing each mean score by the number of items used to calculate the end point score, which allowed the results to remain in the (0–8) units of the original scale. These models were adjusted for baseline CAPS-SX_17_ score and study protocol. Both last observation carried forward (LOCF) and observed case analyses (OC) were performed. In addition to the ANCOVA analysis of the change from baseline score on the unit-standardized CAPS-SX_17_, three transformations were conducted on the CAPS-SX_17_. The first created separate analyses of the original unit-standardized CAPS-SX_17_ for each DSM-IV category (i.e., reexperiencing, avoidance/numbing, and hyperarousal). The second set of transformations created separate analyses for each of the three factors, by averaging only the items that loaded significantly in each of the factors. The third transformation represented factor-weighted adjustments of CAPS-SX_17_, which was obtained by multiplying factor scoring coefficients for each of the CAPS-SX_17_ items before summation.

## Results

### Confirmatory factor analysis

The CFA demonstrated a significant lack of fit for the DSM-IV three-factor PTSD symptom structure in the pooled sample, as well as in the individual trials. The RMSEA criteria (values of 0.05 and 0.06 vs. recommended value <0.05), and Bentler–Bonett Normed Fit Index (value of 0.58 and 0.74 vs. a recommended value of >0.90) in the pooled sample suggested that the EFA was warranted.

**Figure 1:**
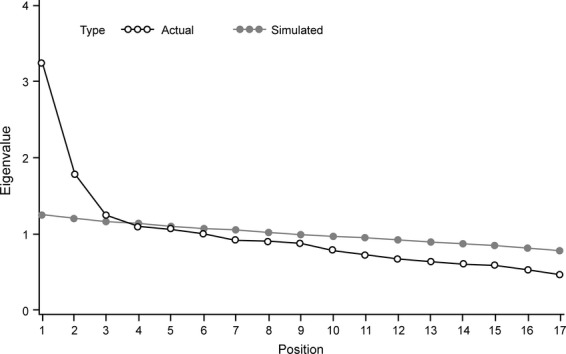
Scree plot of eigenvalues (from reduced correlation matrix) by number of factors. Parallel Analysis – Median Simulated Eigenvalues (17 variables, 1000 iterations, and 860 observations).

The polychoric correlation structure for the pooled studies (Table 3), the scree plot with Horn's parallel analysis (Fig. 1), and SBC goodness-of-fit test from the maximum likelihood factor analysis suggested a three-factor structure. The SBC has the largest absolute value and is the best fit for the three-factor structure (285), with slightly smaller values for two- (236) and four-factor (279) structures. The same analyses were performed with the individual study data, as well as additional analyses that used the pooled Pearson correlation matrix for normally distributed data, all of which produced results that were similar to those described above.

**Table 3:** Factor analysis rotated factor loading for three factors from EFA of polychoric correlation matrix with ML factor extraction and oblique (promax) rotation methods

		Factor 1			Factor 2			Factor 3		
				
	CAPS-SX_17_ item	US	Int'l	Pooled	US	Int'l	Pooled	US	Int'l	Pooled
Reexperiencing	1. Intrusive recollections	**0.75** *	**0.62** *	**0.70** *	0.21	0.14	0.20	−0.04	0.13	0.00
	2. Distressing dreams	0.34	**0.38** **	**0.37** *	0.15	0.02	0.10	0.24	0.10	0.13
	3. Feeling events recurring	**0.47** *	**0.53** *	**0.53** *	0.06	0.16	0.07	0.09	0.04	0.06
	4. Distress at exposure to cues	**0.65** *	**0.70** *	**0.67** *	0.08	0.13	0.08	0.12	0.03	0.13
	5. Reactivity on exposure to cues	**0.50** *	**0.71** *	**0.58** *	0.02	0.10	0.02	0.22	0.03	0.20
Avoidance/Numbing	6. Avoidance of thoughts	**0.37** **	0.32	**0.34** **	0.22	0.16	0.22	0.27	**0.53** *	0.31
	7. Avoidance of activities	0.22	0.25	0.22	0.23	0.22	0.26	0.35	**0.67** *	**0.39** **
	8. Inability to recall trauma	0.02	−0.05	−0.01	0.07	−0.01	0.07	0.18	0.19	0.14
	9. Diminished interest	0.07	0.18	0.11	**0.63** **	**0.67** *	**0.64** *	0.23	−0.04	0.17
	10. Detachment/estrangement	0.07	0.11	0.06	**0.77** *	**0.66** *	**0.75** *	0.07	0.13	0.12
	11. Restricted range of affect	0.08	0.06	0.07	**0.68** *	**0.65** *	**0.66** *	0.06	0.07	0.12
	12. Sense of foreshortened future	0.22	0.30	0.26	0.24	0.37	0.20	0.18	0.07	0.21
Hyperarousal	13. Difficulty falling/staying asleep	0.13	0.29	0.16	0.30	0.19	0.29	0.32	0.08	0.22
	14. Irritability/outbursts of anger	0.18	0.29	0.20	0.24	0.24	0.20	0.19	0.08	0.26
	15. Difficulty concentrating	0.22	0.08	0.14	0.33	**0.46** *	**0.39** *	0.07	0.12	0.12
	16. Hypervigilance	0.06	0.19	0.08	0.09	0.29	0.14	**0.54** *	0.22	**0.54** *
	17. Exaggerated startle response	0.24	0.29	0.27	−0.03	0.22	−0.01	**0.59** *	0.33	**0.54** *

CAPS-SX_17_, 17-item Clinician-Administered PTSD Scale; EFA, exploratory factor analysis; Int'l, international; ML, maximum likelihood; PTSD, posttraumatic stress disorder.

* Lower 95% confidence limit ≥0.30.

** Lower 95% confidence limit ≥0.25.

Therefore, the EFA suggests a three-factor structure; the first two factors loaded on the same items for both studies and the third factor loaded on different items for each study. Items with factors that loaded with a 95% CI ≥0.30 were considered to load highly and significantly on the corresponding factor (Table 3). Factor 1 comprised primarily reexperiencing symptoms, with the highest loading symptoms for items 1 (intrusive recollections), 3 (acting or feeling as if events were recurring), 4 (distress at exposure to trauma cues), and 5 (physiological reactivity on exposure to cues), and potentially item 2 (distressing dreams) and 6 (avoidance of thoughts). Factor 2 mainly consisted of mood and cognitive symptoms, including items 9 (diminished interest), 10 (detachment/estrangement), and 11 (restricted range of affect) and potentially 15 (difficulty concentrating), which loaded highly in the international study but not the US study. For the US study, factor 3 mainly consisted of hyperarousal symptoms: 16 (hypervigilance) and 17 (exaggerated startle response). For the international study, factor 3 mainly consisted of avoidance symptoms: items 6 (avoidance of thoughts, feelings, or conversations) and 7 (avoidance of activities, places, or people). In the rejected four-factor model, arousal and avoidance separated into two different factors. Based on the present data, items 8 (inability to recall important aspect of trauma), 12 (sense of foreshortened future), 13 (difficulty with sleep), and 14 (irritability or outbursts of anger) did not meet the criteria for clear inclusion in any factor.

### Treatment effect analysis

After 12 weeks of treatment with venlafaxine ER or placebo, the original analyses produced an adjusted effect size for the mean treatment difference of −0.32 (*P* < 0.001 vs. placebo; LOCF analysis) (Table 4). Analysis of individual DSM-IV symptom categories (i.e., reexperiencing, avoidance/numbing, or hyperarousal) also produced significant treatment effects: –0.25 (*P* = 0.002), –0.30 (*P* < 0.001), and –0.28 (*P* = 0.001), respectively (Table 5). The three new groupings based on the EFA (reexperiencing [items 1–5]; altered mood/cognition [items 9, 10, 11, and 15]; and avoidance/arousal [items 6, 7, 16, and 17]) produced comparable results: −0.25 (*P* = 0.002), −0.28 (*P* < 0.001), and −0.25 (*P* = 0.001), respectively (Table 6). Compared with unweighted item sums for the suggested factors, factor-weighted adjustment produced a greater effect size (factor 1, −0.27 vs. −0.25; factor 2, −0.30 vs. −0.28; and factor 3, −0.29 vs. −0.25; Tables 6 and 7). Results from the OC analyses were similar.

**Table 4:** Treatment effect on original CAP-SX_17_*, averaged over all items, and each of the original three groupings (LOCF analysis)

Population	Treatment group (*n* at baseline/week 12)	Baseline, mean (SD)	Week 12, mean (SD)	Adjusted mean change**(SD)	Adjusted effect size**(adjusted mean chg/SD)	*P-*value**
Pooled studies	Venlafaxine ER (*n* = 339/324)	4.9 (0.9)	2.2 (1.7)	−2.6 (0.1)	−0.315	<0.001
	Placebo (*n* = 346/332)	4.8 (0.9)	2.7 (1.7)	−2.1 (0.1)		
Study 735	Venlafaxine ER (*n* = 179/171)	4.9 (0.9)	2.5 (1.8)	−2.5 (0.1)	−0.265	0.015
	Placebo (*n* = 179/170)	4.8 (0.9)	2.8 (1.8)	−2.0 (0.1)		
Study 786	Venlafaxine ER (*n* = 160/153)	4.8 (0.9)	2.0 (1.5)	−2.8 (0.1)	−0.397	<0.001
	Placebo (*n* = 167/162)	4.9 (0.9)	2.6 (1.7)	−2.2 (0.1)		

ANCOVA, analysis of covariance; CAPS-SX_17_, 17-item Clinician-Administered PTSD Scale; ER, extended release; LOCF, last observation carried forward; PTSD, posttraumatic stress disorder; SD, standard deviation.

* CAPS-SX_17_ = (item 1 + ··· + item 17)/17.

** From ANCOVA model: CAPS-SX_17_ chg = baseline CAPS-SX_17_ + treatment + pooled site.

**Table 5:** Treatment effect on each of the original three groupings for CAP-SX_17_, averaged over items in category (pooled and LOCF analysis)

Factor number	DSM-IV category	Treatment group (*n* at baseline/week 12)	Baseline, mean (SD)	Week 12, mean (SD)	Adjusted mean change* (SD)	Adjusted effect size* (adjusted mean chg/SD)	*P*-value*
1	Reexperiencing (items 1–5)	Venlafaxine ER (*n* = 339/324)	4.8 (1.3)	2.0 (1.9)	−2.9 (0.1)	−0.249	0.002
		Placebo (*n* = 346/332)	4.7 (1.4)	2.4 (2.0)	−2.3 (0.1)		
2	Avoidance/Numbing (items 6–12)	Venlafaxine ER (*n* = 339/324)	4.7 (1.2)	2.2 (1.9)	−2.6 (0.1)	−0.295	<0.001
		Placebo (*n* = 346/332)	4.8 (1.1)	2.7 (1.9)	−2.1 (0.1)		
3	Hyperarousal (items 13–17)	Venlafaxine ER (*n* = 339/324)	5.1 (1.1)	2.6 (1.9)	−2.5 (0.1)	−0.284	<0.001
		Placebo (*n* = 346/332)	5.0 (1.2)	3.0 (1.9)	−2.0 (0.1)		

ANCOVA, analysis of covariance; CAPS-SX_17_, 17-item Clinician-Administered PTSD Scale; DSM-IV, *Diagnostic and Statistical Manual of Mental Disorders* (Fourth Edition); ER, extended release; LOCF, last observation carried forward; PTSD, posttraumatic stress disorder; SD, standard deviation.

Factor 1-summed CAPS-SX_17_ = (item 1 + item 2 + item 3 + item 4 + item 5)/5.

Factor 2-summed CAPS-SX_17_ = (item 6 + item 7 + item 8 + item 9 + item 10 + item 11 + item 12)/7.

Factor 3-summed CAPS-SX_17_ = (item 13 + item 14 + item 15 + item 16 + item 17)/5.

* From ANCOVA model: CAPS-SX_17_ chg = baseline CAPS-SX_17_ + treatment + pooled site.

**Table 6:** Treatment effect on each of the three new factor-summed CAP-SX_17_, averaged over items in category* (pooled and LOCF analysis)

Factor number	New category	Treatment group (*n* at baseline/week 12)	Baseline, mean (SD)	Week 12, mean (SD)	Adjusted mean change** (SD)	Adjusted effect size** (adjusted mean chg/SD)	*P*-value**
1	Reexperiencing (items 1–5)	Venlafaxine ER (*n* = 339/324)	4.8 (1.3)	2.0 (1.9)	−2.8 (0.1)	−0.249	0.002
		Placebo (*n* = 346/332)	4.7 (1.4)	2.4 (2.0)	−2.3 (0.1)		
2	Altered mood/Cognition (items 9, 10, 11, and 15)	Venlafaxine ER (*n* = 339/324)	5.3 (1.3)	2.5 (2.2)	−2.8 (0.1)	−0.277	<0.001
		Placebo (*n* = 346/332)	5.2 (1.3)	3.0 (2.3)	−2.2 (0.1)		
3	Avoidance/Arousal (items 6, 7, 16, and 17)	Venlafaxine ER (*n* = 339/324)	4.9 (1.4)	2.4 (2.0)	−2.6 (0.1)	−0.252	0.001
		Placebo (*n* = 346/332)	5.0 (1.4)	2.8 (2.1)	−2.1 (0.1)		

ANCOVA, analysis of covariance; CAPS-SX_17_, 17-item Clinician-Administered PTSD Scale; ER, extended release; LOCF, last observation carried forward; PTSD, posttraumatic stress disorder; SD, standard deviation.

Factor 1-summed CAPS-SX_17_ = (item 1 + item 2 + item 3 + item 4 + item 5)/5.

Factor 2-summed CAPS-SX_17_ = (item 9 + item 10 + item 11 + item 15)/4.

Factor 3-summed CAPS-SX_17_ = (item 6 + item 7 + item 16 + item 17)/4.

* Each factor-summed CAPS-SX_17_ category is based on significant factor loadings.

** From ANCOVA model: CAPS-SX_17_ chg = baseline CAPS-SX_17_ + treatment + pooled site.

**Table 7:** Treatment effect on each of the new factor-weighted CAP-SX_17_, averaged over all items* (pooled and LOCF analysis)

Factor number	Treatment group (*n* at baseline/week 12)	Baseline, mean (SD)	Week 12, mean (SD)	Adjusted mean change (SD)	Adjusted effect size (adjusted mean chg/SD)	*P-*value**
1	Venlafaxine ER (*n* = 339/324)	7.1 (1.5)	3.1 (2.6)	−4.0 (0.1)	−0.267	<0.001
	Placebo (*n* = 346/332)	7.1 (1.6)	3.7 (2.7)	−3.4 (0.1)		
2	Venlafaxine ER (*n* = 339/324)	7.0 (1.6)	3.3 (2.8)	−3.7 (0.1)	−0.296	<0.001
	Placebo (*n* = 346/332)	6.9 (1.6)	4.0 (2.8)	−2.9 (0.1)		
3	Venlafaxine ER (*n* = 339/324)	6.6 (1.7)	3.2 (2.5)	−3.5 (0.1)	−0.290	<0.001
	Placebo (*n* = 346/332)	6.7 (1.6)	3.9 (2.6)	−2.8 (0.1)		

CAPS-SX_17_, 17-item Clinician-Administered PTSD Scale; ER, extended release; SD, standard deviation.

* Each factor-weighted CAPS-SX_17_ is calculated by using the factor scoring coefficients as weights on each of the CAPS-SX_17_ item values. Then it is averaged over all items by dividing by 17.

** Analysis of covariance.

## Discussion

Although the DSM-IV conceptualizes PTSD in terms of three symptom clusters, a large and diverse body of data exists suggesting other possible PTSD symptom structures. The most common are four-factor models, although these often include reexperiencing, avoidance, and arousal symptom clusters (Asmundson et al. 2000; Amdur and Liberzon 2001; Baschnagel et al. 2005; McWilliams et al. 2005). Fewer three-factor models have been reported; however, Foa et al. (1995) performed a principal components factor analysis of assault victims that yielded a three-factor structure: arousal/avoidance, numbing, and intrusion. In line with the majority of the data, a four-factor symptom structure is incorporated into the DSM-5 diagnostic criteria for PTSD: (1) reexperiencing, (2) avoidance, (3) arousal and reactivity, and (4) negative alterations in mood and cognition (Friedman et al. 2011). This analyses are at least partially supportive of this approach, having revealed symptom clusters that include reexperiencing, altered mood and cognition, and avoidance/arousal (with avoidance in the international study and arousal in the US study). For both the three-factor DSM-IV and three-factor EFA models of PTSD symptom structures, the current analyses in a large, pooled group of patients with PTSD demonstrated a significantly greater response to venlafaxine versus placebo on all symptom clusters.

Across studies, including factor analyses, conducted in patients with PTSD, there is diversity in the type of populations studied (e.g., male veterans, female psychiatric outpatients), types of trauma (e.g., automobile accidents, rape, exposure to combat), and the assessment tools used (e.g., CAPS-SX_17_, Impact of Event Scale [Horowitz et al. 1979]). It is notable that even within the pooled population assessed here, differences in trauma type were observed between the two studies. Specifically, in the internationally conducted study, the incidence of childhood sexual abuse (1%) (Davidson et al. 2006a) was lower than that in the US study (15%) (Davidson et al. 2006b), which may be attributable to cultural variations associated with discussing traumatic events. The diversity of PTSD patients is a primary limitation of this and other conducted studies. In addition, the criteria used to select a study population for a clinical trial, which generally exclude patients with comorbid psychiatric and substance use disorders, may have created a population that is not representative of PTSD patients in the general population.

The often heterogeneous response to antidepressant pharmacotherapy has led to a questioning of the efficacy of such agents for treating PTSD. The review conducted by the National Institute for Health and Clinical Excellence in the United Kingdom used an a priori definition of clinical significance as an effect size of 0.5, and found that few trials met this threshold (National Institute for Clinical Excellence 2005). Similarly, an Institute of Medicine report, which reviewed available treatments for PTSD, suggested that the data from studies assessing the efficacy of pharmacotherapy are inadequate to demonstrate consistent efficacy. The report argued that the characteristics of and variability among industry-sponsored clinical trials—which use study populations that exclude certain patient types (e.g., substance abusers), have high rates of attrition, and have different methods for addressing missing data—make it hard to generalize their results to the larger patient population (Committee on Treatment of Posttraumatic Stress Disorder 2008). On the other hand, the Cochrane meta-analysis of PTSD treatments found that pharmacotherapy, in particular the selective serotonin reuptake inhibitors, produces clinically and statistically significant improvements in PTSD symptomatology (Stein et al. 2006b). The serotonin–norepinephrine reuptake inhibitor, venlafaxine ER, also has empirically demonstrated efficacy in exerting a statistically and clinically significant treatment response in the primary published studies of these data sets (Davidson et al. 2006a,b) and in a subsequent CAPS-SX_17_ individual item analysis (Stein et al. 2009), and the data here provide additional information on the efficacy of this agent.

One possible explanation of the observed variability in treatment outcomes in PTSD patients is that there are different psychobiological mechanisms that mediate different symptoms. Theories that seek to explain the neurobiological processes underlying PTSD symptomatology have suggested that noradrenergic hyperactivity plays a significant role. Specifically, innervations of noradrenaline from the locus coeruleus to the amygdala, prefrontal cortex, and hippocampus have been linked to the development of conditioned fear responses, which can produce chronic hyperarousal, reexperiencing symptoms, and, in turn, may lead to avoidance behaviors and emotional numbing (Charney et al. 1993). At the same time, serotonin may also play a key role in PTSD, either directly or indirectly, by regulating the activity of noradrenaline (Newport and Nemeroff 2000). Venlafaxine ER blocks the reuptake of both noradrenaline (norepinephrine) and serotonin, which may explain the observed improvements in a range of different symptom clusters. Future research should seek to further clarify the relationship between the neurochemical correlates of PTSD symptomatology by assessing the effect of available treatment options, possibly those with different mechanisms of action, on identified symptom clusters. Performing such analyses may further the understanding of PTSD and lead to improvements in the treatment options available to patients.

This analysis has a number of strengths, including a large and diverse sample size and data pooled from patients treated in a randomized, double-blind design. However, it is important to emphasize a number of limitations. First, as noted above, patients who are enrolled in clinical trials differ from the general population of PTSD patients in important ways, and within each trial there may be further particularities, such as the set of traumas to which subjects were exposed. Second, there was insufficient power to analyze the response of symptom clusters to sertraline treatment (a sertraline arm was included in only one of the studies). Third, because no actual assessment of neurotransmitter activity was conducted, any explanation of how these results relate to the mechanism of action of venlafaxine ER is speculative. Despite these limitations and the preliminary nature of these analyses, the results of the current factor analysis, in the context of the treatment response analysis, support the efficacy of venlafaxine ER for improving all PTSD symptom clusters that are relevant to this patient population. Additional work is needed to confirm the factor structure found here in more representative samples, to determine the underlying psychobiological mechanisms of PTSD symptom factors, and to determine whether these have a differential treatment response.

## Conclusions

This factor analysis of PTSD symptoms suggests an alternate three-factor model that differs from the three-factor model described in the DSM-IV. The data here are consistent with a literature that has failed to confirm the three-factor structure of DSM-IV PTSD, and that has suggested that key symptom clusters in PTSD are reexperiencing, avoidance, arousal, and negative changes in mood and cognition. Furthermore, these analyses provide additional support for the efficacy of venlafaxine ER for treating PTSD by demonstrating a significant treatment effect on the symptoms in the DSM-IV three-factor model and the newly identified three-factor model.

## References

[b1] Amdur RL, Liberzon I (2001). The structure of posttraumatic stress disorder symptoms in combat veterans: a confirmatory factor analysis of the impact of event scale. J. Anxiety Disord.

[b2] American Psychiatric Association (1994). Posttraumatic stress disorder. Diagnostic and statistical manual of mental disorders.

[b3] Asmundson GJ, Frombach I, McQuaid J, Pedrelli P, Lenox R, Stein MB (2000). Dimensionality of posttraumatic stress symptoms: a confirmatory factor analysis of DSM-IV symptom clusters and other symptom models. Behav. Res. Ther.

[b4] Baschnagel JS, O'Connor RM, Colder CR, Hawk LW (2005). Factor structure of posttraumatic stress among Western New York undergraduates following the September 11th terrorist attack on the World Trade Center. J. Trauma. Stress.

[b5] Blake DD, Weathers FW, Nagy LM, Kaloupek DG, Gusman FD, Charney DS (1995). The development of a Clinician-Administered PTSD Scale. J. Trauma. Stress.

[b6] Buckley TC, Blanchard EB, Hickling EJ (1998). A confirmatory factor analysis of posttraumatic stress symptoms. Behav. Res. Ther.

[b7] Charney DS, Deutch AY, Krystal JH, Southwick SM, Davis M (1993). Psychobiologic mechanisms of posttraumatic stress disorder. Arch. Gen. Psychiatry.

[b8] Committee on Treatment of Posttraumatic Stress Disorder (2008). Treatment of posttraumatic stress disorder: an assessment of the evidence.

[b9] Davidson JR (2006). Pharmacologic treatment of acute and chronic stress following trauma: 2006. J. Clin. Psychiatry.

[b10] Davidson J, Baldwin D, Stein DJ, Kuper E, Benattia I, Ahmed S (2006a). Treatment of posttraumatic stress disorder with venlafaxine extended release: a 6-month randomized controlled trial. Arch. Gen. Psychiatry.

[b11] Davidson J, Rothbaum BO, Tucker P, Asnis G, Benattia I, Musgnung JJ (2006b). Venlafaxine extended release in posttraumatic stress disorder: a sertraline- and placebo-controlled study. J. Clin. Psychopharmacol.

[b12] Elhai JD, Ford JD, Ruggiero KJ, Christopher Frueh B (2009). Diagnostic alterations for post-traumatic stress disorder: examining data from the National Comorbidity Survey Replication and National Survey of Adolescents. Psychol. Med.

[b13] Fabrigar LR, Visser PS, Browne MW (1997). Conceptual and methodological issues in testing the circumplex structure of data in personality and social psychology. Pers. Soc. Psychol. Rev.

[b14] Fabrigar LR, Wegener DT, MacCallum RC, Strahan EJ (1999). Evaluating the use of exploratory factor analysis in psychological research. Psychol. Methods.

[b15] Foa EB, Riggs DS, Gershuny BS (1995). Arousal, numbing, and intrusion: symptom structure of PTSD following assault. Am. J. Psychiatry.

[b16] Friedman MJ, Resick PA, Bryant RA, Brewin CR (2011). Considering PTSD for DSM-5. Depress. Anxiety.

[b17] Gaffney M (2003). Factor analysis of treatment response in posttraumatic stress disorder. J. Trauma. Stress.

[b18] Horowitz M, Wilner N, Alvarez W (1979). Impact of Event Scale: a measure of subjective stress. Psychosom. Med.

[b19] Hu L-T, Bentler P (1999). Cutoff criteria for fit indexes in covariance structure analysis: conventional criteria versus new alternatives. Struct. Equ. Model.

[b20] King DW, Leskin GA, King LA, Weathers FW (1998). Confirmatory factor analysis of the clinician-administered PTSD scale: evidence for the dimensionality of posttraumatic stress disorder. Psychol. Assess.

[b21] Mataix-Cols D, Rauch SL, Manzo PA, Jenike MA, Baer L (1999). Use of factor-analyzed symptom dimensions to predict outcome with serotonin reuptake inhibitors and placebo in the treatment of obsessive-compulsive disorder. Am. J. Psychiatry.

[b22] McWilliams LA, Cox BJ, Asmundson GJ (2005). Symptom structure of posttraumatic stress disorder in a nationally representative sample. J. Anxiety Disord.

[b23] National Institute for Clinical Excellence (2005). Post-traumatic stress disorder (PTSD): the management of PTSD in adults and children in primary and secondary care.

[b24] Newport DJ, Nemeroff CB (2000). Neurobiology of posttraumatic stress disorder. Curr. Opin. Neurobiol.

[b25] North CS, Suris AM, Davis M, Smith RP (2009). Toward validation of the diagnosis of posttraumatic stress disorder. Am. J. Psychiatry.

[b26] Stein DJ, Andersen EW, Lader M (2006a). Escitalopram versus paroxetine for social anxiety disorder: an analysis of efficacy for different symptom dimensions. Eur. Neuropsychopharmacol.

[b27] Stein DJ, Ipser JC, Seedat S (2006b). Pharmacotherapy for post traumatic stress disorder (PTSD). Cochrane Database Syst. Rev.

[b28] Stein DJ, Andersen EW, Overo KF (2007). Response of symptom dimensions in obsessive-compulsive disorder to treatment with citalopram or placebo. Rev. Bras. Psiquiatr.

[b29] Stein DJ, Pedersen R, Rothbaum BO, Baldwin DS, Ahmed S, Musgnung J (2009). Onset of activity and time to response on individual CAPS-SX17 items in patients treated for post-traumatic stress disorder with venlafaxine ER: a pooled analysis. Int. J. Neuropsychopharmacol.

